# Advances in Spray-Drying and Freeze-Drying Technologies for the Microencapsulation of Instant Tea and Herbal Powders: The Role of Wall Materials

**DOI:** 10.3390/foods14030486

**Published:** 2025-02-03

**Authors:** Júlia Mazár, Krisztina Albert, Zoltán Kovács, András Koris, Arijit Nath, Szilvia Bánvölgyi

**Affiliations:** Department of Food Process Engineering, Institute of Food Science and Technology, Hungarian University of Agriculture and Life Sciences, H-1118 Budapest, Hungary; mazar.julia@phd.uni-mate.hu (J.M.); kovacs.zoltan.andras@uni-mate.hu (Z.K.); arijit.nath@uni-mate.hu (A.N.);

**Keywords:** microencapsulation, spray-drying, freeze-drying, wall material, core material, instant herbal powder, instant tea powder

## Abstract

The microencapsulation of tea and herbal extracts is gaining considerable attention in the food industry, particularly in the production of instant powders. This review examines the application of spray-drying and freeze-drying technologies for the encapsulation of bioactive compounds, focusing on the role of wall materials. Over the past two decades, carbohydrate-based (e.g., maltodextrin), gum-based (e.g., gum Arabic), and protein-based (e.g., whey protein isolate) materials have been widely used due to their impact on sensory properties, stability, protection of bioactive compounds, and other critical attributes of encapsulated products. Despite their widespread use, these materials have distinct advantages and limitations, such as cost, availability, and compatibility with different extracts. This review provides a comprehensive analysis of their physical and chemical properties, examines alternative and emerging wall materials (e.g., beta-cyclodextrin, sodium alginate, and inulin), and highlights the potential of combining different materials to optimise encapsulation outcomes. It also identifies current research gaps and future directions to improve the efficacy and quality of encapsulated tea and herbal powders.

## 1. Introduction

The consumption and use of tea and herbal infusions date back to ancient times, with evidence of use before the Common Era. Since then, tea has become one of the most widely consumed beverages globally, enjoyed by approximately two-thirds of the world’s population [1]. It is consumed not only for its sensory qualities but also for its potential role in disease prevention, largely attributed to its high antioxidant content [2,3]. Recent studies indicate a resurgence in the consumption of products derived from medicinal plants, reflecting a notable renaissance in their use [4], emphasizing the renewed interest in the therapeutic potential of these botanical resources. The therapeutic and healing properties of herbal teas have led to their widespread consumption across various regions worldwide. Aromatic herbal blends have become an integral component of the tea industry. Recent years have seen a significant rise in demand, as evidenced by data from the Passport^®^ database. It is anticipated that this trend will endure, especially in the wake of the global pandemic caused by the novel strain of coronaviruses (SARS-CoV-2), which has served to emphasise the significance of preventative health measures [5].

The industrial processing of plant materials in the production of filter tea generates a significant volume of by-products and waste, accounting for approximately 10–40% of the initial material. Among these by-products, “herb powder” is of particular importance, defined as plant matter with a particle size smaller than the diameter of tea filter pores. Advances in food industry technology have enabled the transformation of this herb powder into a new, high-quality product: instant tea powder [6]. The production of instant powders is the result of microencapsulation technology, which is currently being developed by a number of researchers. This approach not only facilitates waste reduction within the industry but also enables the production of a new, user-friendly, functional beverage. In recent years, there has been a marked increase in demand for such products, contributing to significant growth in this sector [7,8].

In the course of our research, we conducted a comprehensive review of English-language publications from the past two decades, with a specific focus on the encapsulation of teas and herbal extracts. The bibliographic search was carried out from February 2024 to November 2024, during which we collected the relevant studies using four main scientific databases available: Google Scholar, ResearchGate, ScienceDirect, and Scopus. During the search, specific keywords were used to identify pertinent studies, including “tea powder”, “instant tea powder”, “instant herb powder”, “encapsulation”, “microencapsulation”, “spray-drying”, “freeze-drying”, “wall material”, “maltodextrin”, “β-cyclodextrin”, ”chitosan”, “gum Arabic”, “sodium alginate”, “inulin”, and “whey protein isolate”. We selected review articles, research articles, and book chapters that satisfied two key criteria: first, they had to be published between 2007 and 2024; second, their title or abstract needed to explicitly mention the encapsulation of tea or herbal extracts. The information that was found to be relevant was then used to collect introductory information, definitions, and general overview from the articles and book chapters. The gathered data is systematically presented in the form of diagrams and tables, which will be analyzed in detail in the subsequent sections of this article. This methodology enables an extensive and current examination of the topic, offering a robust foundation for understanding recent developments in encapsulation technologies and wall materials applied to tea and herb instant powder.

The literature includes numerous studies focusing on instant powder, which produces microencapsulation of black tea, green tea, and medicinal teas such as chamomile [5,9,10], sage [9,11], peppermint [5,9,12], lemongrass [9,13,14], yarrow [6,9], hibiscus [15,16,17], rosehip [15], and ginseng tea [8,18] using spray- and freeze-drying microencapsulation techniques. These studies employed various wall materials at different concentrations throughout the drying process and investigated how different wall materials influenced the physical, chemical, and sensory properties of the resulting powders. Wall materials based on polysaccharides, proteins, and lipids are currently used in various areas of the food industry, but specifically in the case of tea and medicinal plants, much less information is available on the effect of different wall materials. In the course of our present research, we aim to provide an overview summarizing the scientific progress of recent years in the field of encapsulation of tea and herbal extracts, which provides an up-to-date overview of the production technologies utilized and relevant information about the currently used wall materials.

## 2. Instant Tea Powder Production

Instant tea, also known as instantly soluble tea, refers to a powdered or granulated product that rapidly dissolves in water to create a tea beverage. The production of instant tea involves extracting the active components from tea leaves, which can include black tea, green tea, or various herbs and medicinal plants. These extracts are then processed into a powder or granule form, facilitating instant solubility in water. Instant teas may be derived from pure extracts, where the tea or herbal material is directly processed without any additives. Alternatively, they can be formulated as a mixture of extracts, which may or may not involve the incorporation of wall materials. Wall materials, such as maltodextrin, gum Arabic, or other polysaccharides, are often used in the encapsulation process. These materials act as protective carriers during spray-drying or other encapsulation techniques, preserving the volatile components of the tea while improving the powder’s ease of use and the aforementioned properties [19,20,21].

The production of instant teas can be regarded as a form of encapsulation, a highly efficient process widely utilized across various industries, including pharmaceuticals, cosmetics, and food production. Encapsulation technology plays a crucial role in preserving active compounds, enhancing stability, and improving the solubility of ingredients, making it an integral method in the development of instant tea products [3]. Additionally, encapsulated tea products provide excellent convenience and precise dosing, ensuring a consistent and high-quality tea experience for consumers. This technology facilitates the encapsulation of active ingredients with an increased concentration in the products, thereby preserving the bioactivity of these substances for a longer period of time, thus ensuring the long-term development of positive physiological effects. By protecting sensitive ingredients from environmental factors such as oxidation, moisture, and light, encapsulation ensures that the active compounds retain their efficacy throughout the product’s shelf life [3,15,22]. As a result, instant tea powders facilitate the standardization and quality control of teas and herbal preparations, ensuring consistent product quality in the future [6,10]. Instant powders offer greater stability and a reduced volume, making them easier to store and transport. This characteristic contributes to lower production and distribution costs [3,6,22]. The method of preparation is also more straightforward and quicker compared to traditional filter teas [3]. At present, instant tea powder is predominantly utilized in the formulation of tea drink mixes, baked goods, snacks, and various other food products that incorporate tea flavors [23,24], but some researchers have already tested its effects and benefits in meat preparations [25,26].

**Figure 1:**
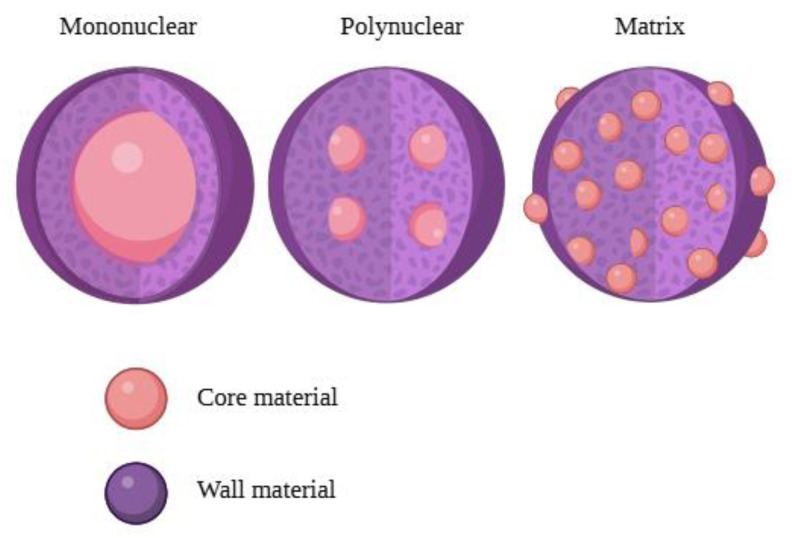
Schematic representation of the types of capsules and their structure: mononuclear (**left**), polynuclear (**middle**), matrix (**right**) (Illustration created with BioRender.com; Adapted from: [33]).

In the production of instant tea powder, the chosen material or combination of materials is encapsulated or coated with another substance or system (see Figure 1). The encapsulated material is referred to as the inner phase, filling [27], active component, or core material [28]. The coating material is called membrane, shell, wall, carrier, or encapsulating material [27,28,29]. In certain instances, the use of an additional wall material may not be required, as the initial material can also perform this function effectively [30,31,32].

Instant tea powders can be produced using various technologies, including spray-drying, contact drying, and freeze-drying [3,10,34]. During the research of Kraujalytė and colleagues [34], in which the volatile compounds and sensory characteristics of black tea produced with different technologies were examined, it was determined that, based on the results of the descriptive sensory analysis, in most cases, no significant difference (*p* > 0.05) was found between the taste characteristics. However, several studies have shown significant differences in the content (antioxidant capacity, acid content, etc.), physical properties (morphological properties, dry matter and ash content, water activity, and color values, etc.), and powder properties (bulk and tapped densities, flowability, cohesion, adsorption, and solubility, etc.) [35,36].

**Figure 2:**
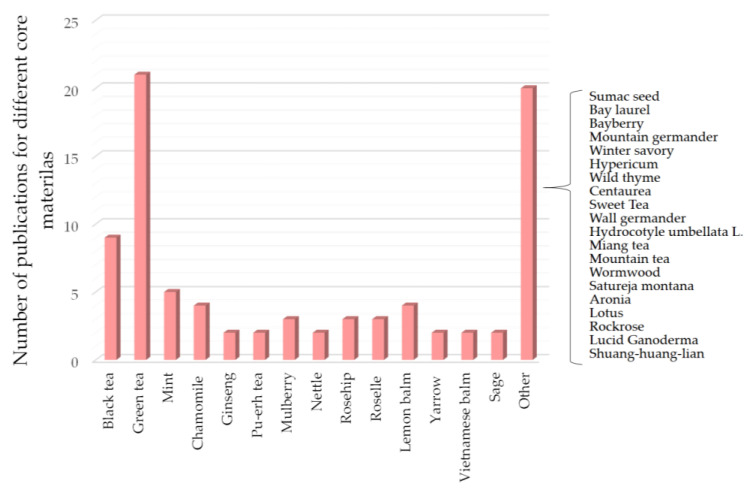
Core materials: the proportion of tea and herbs used as core materials in studies published between 2007 and 2024.

In Figure 2, the summary highlights the various teas and herbal extracts studied for the production of instant powders, focusing predominantly on green tea, which is the most researched. Black tea follows closely in terms of research interest. Although there is growing attention toward the microencapsulation of medicinal teas, the review of existing literature reveals that this area remains underexplored and demands further investigation to optimize the encapsulation processes and assess the benefits of using different wall materials and drying techniques. This underscores the necessity for continued research to improve both the efficiency and quality of instant tea powders derived from medicinal plants and herbal extracts.

### 2.1. Spray-Drying Technology

One of the most widely reported technologies in the literature [20,37,38] and the most commonly used drying method for producing instant products from tea extracts is spray-drying [3,20]. Spray-drying is a highly effective method of producing complex particles or capsules. It has been used for decades to encapsulate various food ingredients, including flavours, lipids, and carotenoids [27,39]. The primary objective of this technology is to eliminate moisture from wet materials utilizing warm air [40]. The spray-drying process consists of three main stages: first, atomization, where the liquid is turned into fine droplets; second, the conversion of these droplets into solid particles; and third, the collection of the particles. However, some sources describe the process in four or five stages instead [27,41,42].

**Figure 3:**
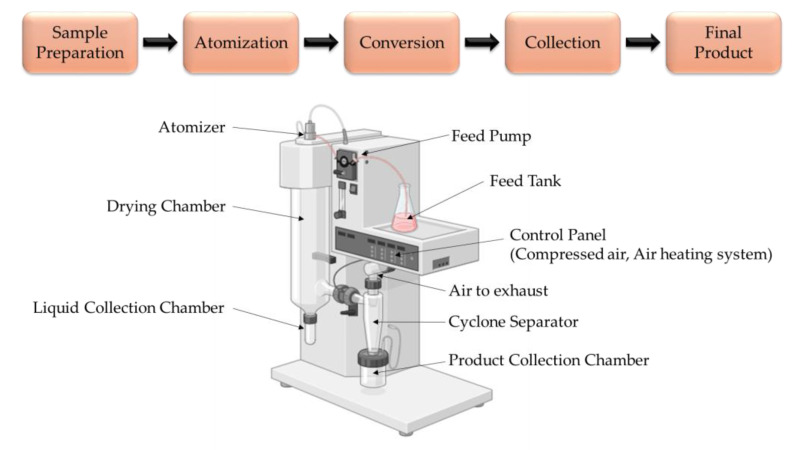
Process diagram of spray-drying (Illustration created with BioRender.com; Adapted from: [42]).

Figure 3 illustrates the process diagram of spray-drying. In the initial stage, the emulsion or extract is introduced into a sprayer, where the supplied liquid is atomized into a fine mist of droplets. Then, the droplets are sent to a drying gas chamber, where intense moisture evaporation occurs under the influence of hot air, resulting in the formation of a polymer capsule shell, which is deposited on the surface of the particles [6,28,37,42]. Finally, the dried particles are separated from the drying medium using a suitable device, typically a cyclone, and collected in a separate container [28,37,42]. The drying process occurs rapidly, preventing heat damage to the product even at elevated temperatures [43].

This technique enables the production of particles ranging from 10 to 100 μm in size, which qualifies as microencapsulation [28]. The moisture content and water activity values of the spray-dried powder were statistically lower than those of the freeze-dried powder (*p* < 0.05) [36]. The average water activity of instant teas produced via spray-drying typically ranges from 0.1 to 0.57 [10,11,23,30,36], placing them in the category of low-moisture food products. These foods are considered microbiologically stable, as water activity levels below 0.6 disrupt the physiological processes required for cell division [44,45]. Powdered products are solid particulate materials typically ranging in size from 50 to 1000 µm, and can be categorized based on their primary characteristics (intrinsic properties) or secondary characteristics (bulk properties) [46,47]. Spray-drying forms spherical particles, the core material is completely protected, the encapsulation is uniform, and there are no pores on the surface [24]. As demonstrated above, this technology boasts numerous advantages. The procedure can be designed for heavy capacity, with feed rates able to range from a few dekagrams to in excess of 100 tons per hour. Furthermore, the operation can be adapted to continuous and automatic control, and it can be used with both heat-resistant and heat-sensitive products [41].

The characteristics of the final product, specifically instant tea powder, are influenced by various parameters during the microencapsulation process. In spray-drying, key factors impacting the product include the spray flow rate [48], the diameter of the nozzle, the air pressure from the compressor, and the feed temperature [49]. Additionally, both the inlet and outlet air temperatures play significant roles [48,49]. Other elements affecting the properties of the final product, namely the capsules, include the dry matter content, the quality of the materials used, the concentration and viscosity of the feed mixture, as well as its sugar content.

During the spray-drying process, the choice of wall material is a critical factor influencing the properties of instant tea powder. The wall material significantly affects the microstructure of the resulting capsules [28]. Numerous studies have demonstrated that the size, shape, and surface characteristics of the microcapsules are contingent upon the properties of the wall material [50,51,52]. Furthermore, the solidification mechanisms during spray-drying are also influenced by the chemical properties and interactions between the core and the wall material [50]. Table 1 summarizes the research that used spray-drying microencapsulation for tea and herbal extracts

### 2.2. Freeze-Drying Technology

**Figure 4:**
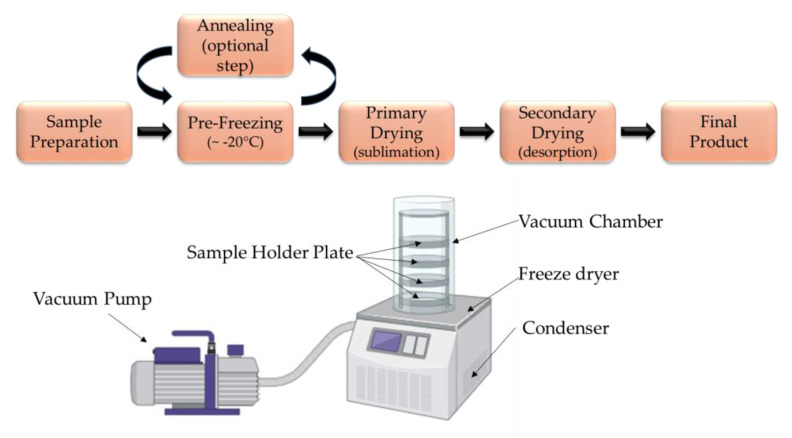
Process diagram of freeze-drying (Illustration created with BioRender.com; Adapted from: [85]).

Freeze-drying is a widely used (food and pharmaceutical industry) and effective method that is also used in microencapsulation. The use of lyophilization in the food industry became widespread at the end of the 20th century. Although expensive and time consuming, it is one of the most effective and gentle drying and preservation methods available today [84,85]. Freeze-drying is a multi-step process that includes pre-freezing (~−20 °C), primary drying (sublimation), secondary drying (desorption), and finally storage [24,86]. During the technology, the feed solution containing the coating and the core material is frozen at a very low temperature (below −40 °C) and dried by sublimation under reduced pressure (<300 Pa) [85,86]. Figure 4 illustrates the process diagram of freeze-drying.

Freeze-drying is suitable for processing compounds sensitive to heat and oxygen [24,87], due to the low temperature and oxygen-deficient environment during the drying process [87]. As a result, this drying method is important for sensitive compounds where the phenol content, bioactivity, appearance, color, texture, aroma, and nutritional value of the food are of great importance [36]. The gentleness of the procedure has already been confirmed by several studies during the examination of different teas, although the results depended to a large extent on the type of tea used. A higher concentration of volatile substances [34] and a higher antioxidant capacity [55] were found in black tea-based instant powder samples produced by freeze-drying compared to instant powders produced by spray-drying. On the other hand, when examining green tea and rosehips, antioxidant capacity and antioxidant content were higher during spray-drying [54,88]. In the case of green tea, it was also observed that freeze-drying improved or preserved the color characteristics and vitamin C content of the product [88].

With this method, particles in the range of 1–150 μm can be produced, which also belongs to the size range corresponding to microencapsulation [24,87,89]. The average water activity of instant teas produced by spray-drying is typically between 0.1 and 0.6 [10,36,54,87]. Their water activation is below 0.6, according to which they also belong to foods with low moisture content. During freeze-drying, sublimation of ice results in smooth, porous, angular particles that result in uneven, irregular encapsulation [24,87,89]. Due to this morphological structure, the core material is much more exposed to external influences than the products produced during spray-drying. Freeze-dried particles show a high brittleness due to the physical characteristics listed above, thanks to which they can be easily broken into fine particles [87]. Relatively better results can be achieved with these particles in terms of flowability, average wettability, and dissolution time [36,90].

Freeze-drying is a simple operation [89], but more expensive than other microencapsulation techniques [91]. The costs of freeze-drying are up to 50 times higher than the costs of spray-drying, and the storage and transportation of the generated particles are extremely expensive; the commercial applicability is also strongly limited by the long processing time [28]. Due to the above, it is used in the food industry only for high-value foods, such as for coffee, spices, and for drying meat and food ingredients. However, various developments and researches are already underway that have the potential to render freeze-drying a more cost-effective and efficient treatment (such as the combination with adsorption, fluidization, and microwaves) [92].

Currently, freeze-drying is not a very common method for the microencapsulation of tea and herb extractions; rather, this technology is used for the encapsulation of various oils. In Table 2, we summarize the research that used freeze-drying microencapsulation for tea and herbal extracts.

## 3. Wall Materials

The wall material serves as a coating for the microencapsulation of bioactive compounds, primarily possessing film-forming properties. Its primary function during the drying process is to safeguard the bioactive compounds within the core material from environmental factors, including exposure to oxygen, moisture, light, heat, and other constituents [15,39,94]. In addition, wall materials play a crucial role in preventing the evaporation of volatile ingredients, allowing the active compounds to be incorporated into a free-flowing powder. This makes the product easier to handle and use in various food applications [94,95]. This is usually concluded with the encapsulation efficiency, which is the difference between the total amount released at the end of the release test and the percentage of compound released at time zero [52]. However, these effects are only significant up to a certain optimal level, after which they may not provide additional benefits [15].

In addition to the previously mentioned factors, the wall material also influences the bulk density and solubility of instant tea powder. Bulk density is a key parameter for powdered products, as a higher density can reduce packaging and transportation costs, while a lower density impacts other properties such as flowability and instant dissolution characteristics. “Solubility” here refers to the powder’s ability to dissolve or form a suspension in water [11]. Şahin-Nadeem and colleagues [11] found that the solubility of instant powders depends on factors such as the air inlet temperature, the type of wall materials, and their concentration.

The selection of the optimal wall material for microencapsulation is a complex process, influenced by various factors such as the intended use and functional requirements of the product, the characteristics of the core material, the encapsulation technique employed, cost considerations, and regulatory approvals from bodies such as the U.S. Food and Drug Administration (FDA) or the European Food Safety Authority (EFSA). According to research by Fang and Bhandari [60], using large amounts of wall materials during the drying process can significantly increase production costs and alter the sensory properties of the final product, particularly its taste, which may lead to consumer dissatisfaction. The challenge of selecting an appropriate wall material is further compounded by the wide variety of materials available, including proteins, carbohydrates, lipids, gums, and cellulose [28], each with distinct properties that can influence the microencapsulation outcome.

The functionality profile of an ideal wall material for spray-drying is characterized by a high degree of complexity. The encapsulating material should exhibit low viscosity, effective emulsifying and film-forming properties, low hygroscopicity, resistance to degradation in the gastrointestinal tract, biodegradability, and solubility in aqueous solvents, as well as being cost-effective and sourced from reliable suppliers [72,94]. However, as each material has its own advantages and limitations, no single wall material meets all these criteria [28]. As a result, different combinations of wall materials are frequently employed to optimize performance and achieve the desired encapsulation properties [28,39].

Additives most commonly used in the spray-drying of teas and herbal products include high molecular weight carbohydrates such as modified starch (of which dextrans and cyclodextrins are the most researched and used), gum Arabic, proteins (especially whey protein isolate (WPI) and sodium caseinate), and polysaccharide-based wall materials such as inulin, alginate, and chitosan. Furthermore, polymers and colloidal silicon dioxide (SiO_2_) are frequently employed to enhance the drying process and product stability [56]. In freeze-drying, based on the research carried out so far, most of the tests have been carried out with maltodextrin or gum Arabic, or no wall material has been used at all.

**Figure 5:**
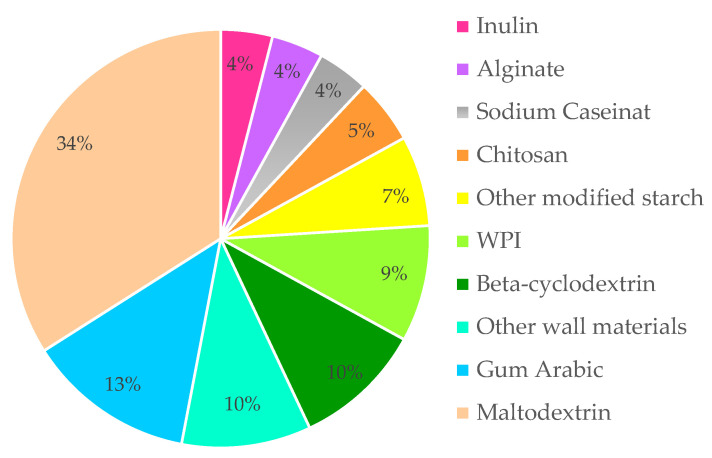
Wall materials used in the microencapsulation of tea and herbs: he proportion of materials used as wall materials in studies published between 2007 and 2024.

As demonstrated in Figure 5, an overview of the wall materials utilized in tea and herb-based microencapsulation research over the course of the past two decades is provided.

### 3.1. Modified Starches

During microencapsulation, different native starches are not the best choice, as their structure is not so stable, their solubility is poor, and their processability is poor. On the other hand, starches are chemical or physical modifications, so modified starches can have much better properties for encapsulation. This group includes maltodextrin, which is the most commonly used wall material in the industry. There is more and more research on beta-cyclodextrin and chitosan, so these substances are worthy of consideration. For this reason, among the modified starches, these three substances will be presented in more detail in the following. However, many other modified starches are also mentioned in the literature, such as modified corn starch [10,51] or sodium octenyl succinate modified starch [69].

#### 3.1.1. Maltodextrin

**Figure 6:**
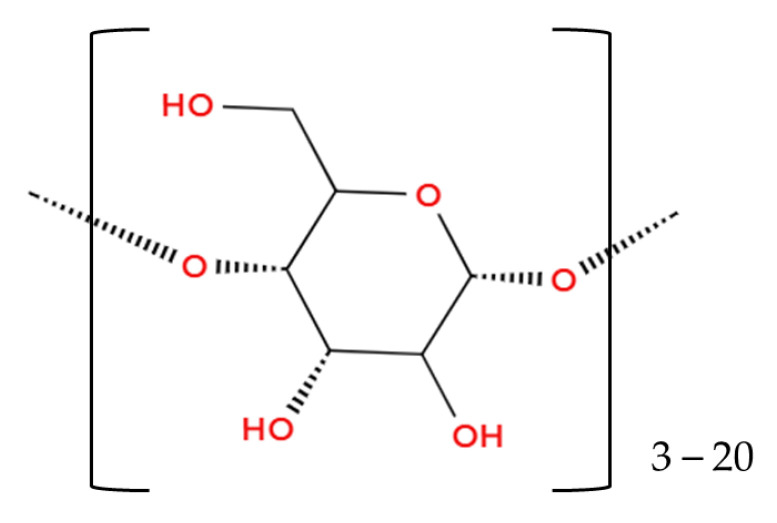
Maltodextrin (Illustration created with MolView.com; Adapted from: [98]).

The use of maltodextrin [(C_6_H_10_O_5_)_n_·H_2_O] (see Figure 6) as a wall material has been a well-established method in food powder production for several decades. Presently, it remains the most commonly employed wall material in the microencapsulation of teas, medicinal herbs, and medicinal plants through spray and freeze-drying, as demonstrated in numerous studies [6,17,30,96,97].

Maltodextrin is produced through acidic and/or controlled enzymatic hydrolysis of starch. It consists of D-glucose polymers connected by α-(1,4) and α-(1,6) glycosidic bonds [99]. The extent of starch polymer hydrolysis is measured by the dextrose equivalent (DE) value, which reflects the content of reducing end groups. This value is inversely proportional to the average degree of polymerization (DP) of the dehydrated glucose units [28,99].

Maltodextrin offers excellent value for money, as it is not only cost-effective [28,58,68] but also possesses numerous advantageous properties, making it an ideal wall material for microencapsulation. It is slightly sweet and nearly tasteless [28,58,99], highly soluble in water, readily dispersible, and exhibits moderate to low solubility in alcohol [99]. Additionally, it has low viscosity at high solids concentrations and is available in various average molecular weights [28]. Generally, maltodextrins are hydrophilic, though this characteristic varies with the dextrose equivalent (DE) value. For instance, maltodextrin with a DE value of 10 is less hydrolyzed, resulting in fewer hydrophilic groups and fewer water bonds, which in turn increases wetting time [30].

The use of maltodextrin in spray and freeze-drying lowers the moisture content of the final product by increasing the total dry matter content. Additionally, it decreases the hygroscopicity of powders, particularly when higher concentrations of wall materials are used. Maltodextrin also enhances the rehydration capacity of dried products, as the less adhesive particles dissolve more easily in water, creating a larger contact surface with the liquid [30]. Increasing maltodextrin concentrations leads to higher powder bulk density, reduced hygroscopicity, and an increased water solubility index, while the water absorption index decreases also found that higher concentrations of maltodextrin can alter the physical properties of the product, influencing solubility and bulk density, and aiding in the formation of particles with a more desirable shape [79,80]. It was also observed that a greater amount of maltodextrin results in smaller, more distinct particles. Mora-Flórez and colleagues [5] further confirmed that using maltodextrin as the primary wall material produces smooth, spherical particles.

The addition of maltodextrin, however, can present drawbacks, such as altering the nutritional value of the product by increasing its carbohydrate content [30]. Furthermore, the retention of flavor compounds tends to decrease when maltodextrin with a higher dextrose equivalent (DE) value is used [28]. The concentration of maltodextrin also affects the chemical composition and levels of bioactive compounds, which are beneficial to health. For instance, higher concentrations of maltodextrin have been shown to reduce total phenolic, total flavonoid, and antioxidant contents [79].

A newer variation of maltodextrin is resistant maltodextrin, a randomly linked alpha-glucoside oligosaccharide with a low glycemic index (around 10% of traditional maltodextrin). While most research has focused on its nutritional benefits, only a few recent studies have explored its use as an encapsulating material in spray and freeze-drying methods [72].

#### 3.1.2. β-Cyclodextrin

**Figure 7:**
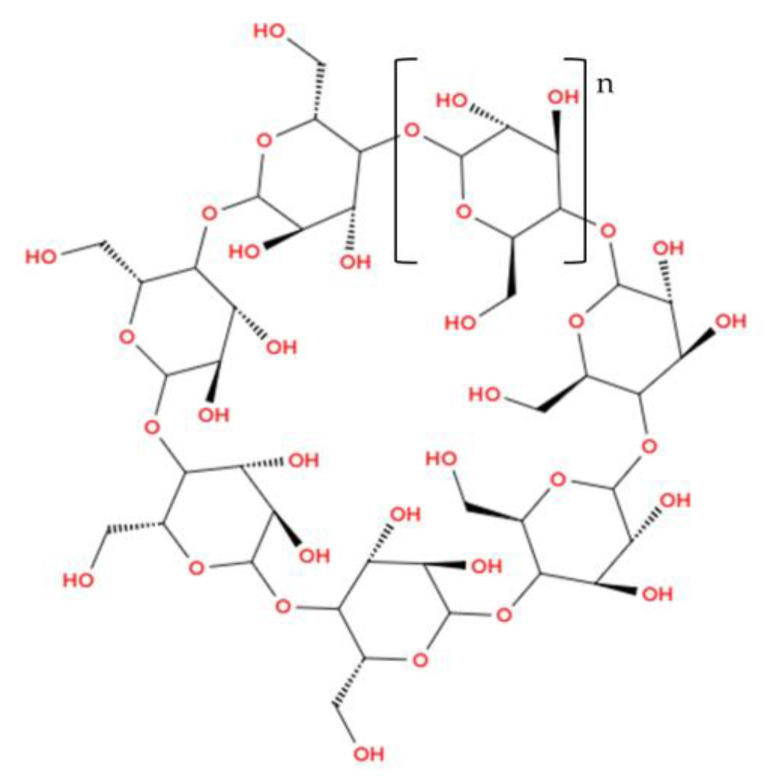
Beta-cyclodextrin (Illustration created with MolView.com; Adapted from: [103]).

β-cyclodextrin [(C_6_H_10_O_5_)_7_] (see Figure 7) is a molecule characterized by both chemical and physical stability, and is produced through the enzymatic modification of starch. Specifically, starch is converted into cyclic dextrins via the action of the enzyme cyclodextrin glucosyltransferase [28,65]. Structurally, β-cyclodextrin is composed of seven glucopyranose units linked by α-(1-4) glycosidic bonds, forming a macrocyclic ring [28,100,101,102]. The spatial configuration of this molecule resembles a truncated cone, with hydroxyl groups oriented toward the exterior of the cavity, making the outer surface polar while the interior cavity remains apolar [99].

The unique structure and physicochemical properties of cyclodextrins allow them to encapsulate molecules of other substances, making them ideal for molecular encapsulation. In an aqueous solution, the weakly apolar cavity of cyclodextrins is occupied by water molecules, which are energetically unfavorable due to polar–apolar interactions. These water molecules can easily be replaced by suitable “guest molecules” that are less polar than water. In this interaction, cyclodextrin acts as the “host molecule,” and the driving force behind complex formation is the displacement of high-enthalpy water molecules with appropriate guest molecules [104].

The additional properties of β-cyclodextrin offer even greater potential when applied as a wall material in encapsulation processes. Not only does it protect the core material from environmental factors, but it also provides protection against degradation caused by temperature and pH fluctuations [24]. Mourtzinos et al. [69], during their research on the encapsulation of lemon balm, confirmed that the hollow structure of β-cyclodextrin protects against the oxidation of extract components. Kalogeropoulos et al. [65], in their study on St. John’s Wort, and Mourtzinos et al. [69], during their research on the encapsulation of lemon balm, confirmed that the hollow structure of β-cyclodextrin protects against the oxidation of extract components. Additionally, β-cyclodextrin improves the shelf life of foods and their bioactive compounds more effectively than maltodextrin and helps to mask or reduce undesirable odors and flavors [59]. According to the research by Şahin-Nadeem et al. [20], gum Arabic and maltodextrin were more effective in reducing aroma loss compared to β-cyclodextrin during the spray-drying of mountain tea water extract. However, they also found that β-cyclodextrin resulted in the highest bulk density among the three wall materials studied.

Another key aspect of microencapsulation is solubility. β-cyclodextrin is effective in enhancing the water solubility of various poorly soluble compounds [69]. During the encapsulation of sage, Şahin-Nadeem et al. [11] observed that β-cyclodextrin provided better quality characteristics, particularly in terms of solubility and turbidity of the produced samples, compared to gum Arabic and maltodextrin. When used as a wall material, β-cyclodextrin improves the water solubility of phenolic compounds during encapsulation [59]. Furthermore, cyclodextrins exhibit self-aggregating properties, although the degree of aggregation depends on the molecular weight of the cyclodextrin and the interaction between the guest and host molecules [24].

#### 3.1.3. Chitosan

**Figure 8:**
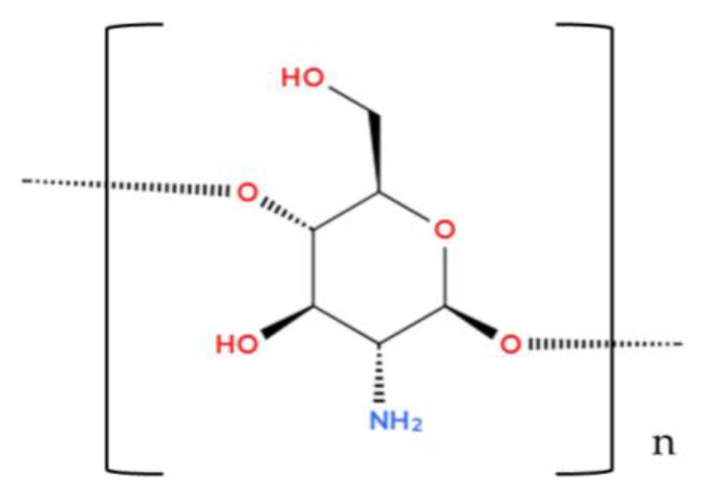
Chitosan (Illustration created with MolView.com; Adapted from: [105]).

Chitosan [(C_6_H_11_NO_4_)_n_] (see Figure 8) is an N-deacetylated derivative of chitin and belongs to linear polysaccharides [57,105]. This biopolymer is one of the most abundant natural substances [52]. A substance found in large quantities in the exoskeletons of crustaceans and some insects [57]. Due to its various properties, chitonase is widely useful in various sectors, such as the pharmaceutical industry (controlled release preparations), agriculture (mold killer), winemaking (facilitates sedimentation and clarification of musts), and the food industry (thickener, stabilizer, and emulsifier) [52,83]. Since its FDA (U.S. Food and Drug Administration) approval, kitolone’s pharmaceutical and food applications have continued to evolve in recent years [106]. However, basic chitonase is not usually used in the food industry, since one of the main disadvantages of this substance is that it does not dissolve in water, only in acidic solutions [52]. For this reason, you prefer to use its chemically modified version, in which hydrophilic functional groups are added, or a low molecular weight, water-soluble substance is created through a depolymerization process [52,57].

Modified chitosan is white, cream-colored, tasteless, odorless in purified form, and soluble in aqueous solutions. This wall material is biodegradable, bio-compatible, non-toxic, and has low cost [83]. Currently, chitosan is mostly used as a wall material in the encapsulation of oils, but more and more researches use it by itself or in combination with other carrier materials for tea and herbs. Estevinho et al. [107] compared chitosan with modified chitosan as a wall material. They found that the modified chitosan had a release time pattern 12 times shorter than chitosan, and the slow release was associated with a rougher capsule surface. Kasapoğlu et al. [40] compared WPI and chitosan during encapsulation of *Rosa pimpinellifolia* extract by spray-drying. Based on the study, chitosan layer protected the heat-sensitive antioxidant effect better than WPI during spray-drying; however, no significant difference was found between the two biopolymers based on their stability during in vitro digestion.

### 3.2. Gums

Gums are natural or artificially produced hydrocolloids that are widely used in various industries due to their special properties. Several natural gums are used in the food industry. Natural sources of gums can be, for example, plant extracts or algae. The tires are also noteworthy from a sustainability point of view, as many of them are biodegradable, environmentally friendly, and come from renewable sources. For the microencapsulation of tea and medicinal herbs, gum Arabic and sodium alginate were used in most cases among the different gums.

#### 3.2.1. Gum Arabic

**Figure 9:**
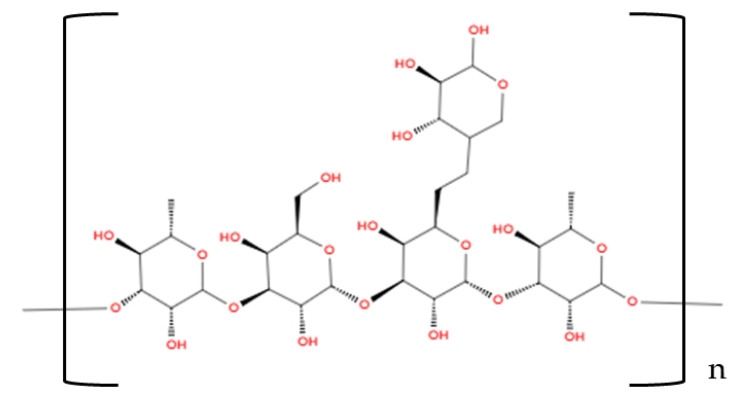
Gum Arabic (Illustration created with MolView.com; Adapted from: [109]).

Gum Arabic is a widely used and applied material in the food industry, serving as a stabilizer, thickener, emulsifier, and bulking agent. The most popular polyphenolic coating material is gum Arabic [89]. It is derived as a dried exudate from the branches and trunks of Acacia Senegal Willdenow or closely related species of the Acacia (*Leguminosae* family). The composition primarily consists of high molecular weight polysaccharides and their calcium, magnesium, and potassium salts. Upon hydrolysis, it yields arabinose, galactose, rhamnose, and glucuronic acid [108] (see Figure 9).

They are white or yellowish-white in color [110], usually tasteless or neutral, but can affect the taste and flavor of food [28,89]. This change is due to hydrocolloid flavors as they reduce sweetness by viscosity and hindered diffusion [28].

In the literature, there are already several studies dealing with gum Arabic as a wall material used in the microencapsulation of tea and medicinal herbs. Green tea [53,74,83], hibiscus [16,70], mountain tea [13], and Miang tea [87] have been used in capsules. It is a versatile wall material, as it has several positive properties in terms of encapsulation. The highest levels of encapsulation efficiency can be achieved in both the spray-drying and freeze-drying processes [13,87]. This wall material has good water solubility, low viscosity in aqueous solutions, and emulsifying properties, and is sensitive, easily decomposable, and can retain water well [28,57]. In addition, the wall material is ideal for the treatment of lipid droplets, as it serves as both a surfactant and a drying matrix, so it can be used to attract the loss of volatile substances with the associated environment. The use of gum Arabic for encapsulating powder helps to avoid the Maillard reaction occurring in the extract, i.e., the caramelization of the sugar remaining in the product, and helps to avoid the powder sticking to the wall of the dryer [110].

However, its use in the food industry is limited; although gum Arabic is considered an excellent wall material, its supply is expensive and limited in availability [58]. As a result, they are looking for alternatives or combinations of this material with other materials. Among the gums, Brea gum, a hydrocolloid extracted from the wood of *Cercidium praecox*, should be such a substitute. It can replace gum Arabic in many applications because of its similar functional properties [111]. In research, it is most commonly combined with maltodextrin, which can effectively replace and supplement gum arabic at levels of 50% or more during encapsulation [13,58].

#### 3.2.2. Sodium Alginate

**Figure 10:**
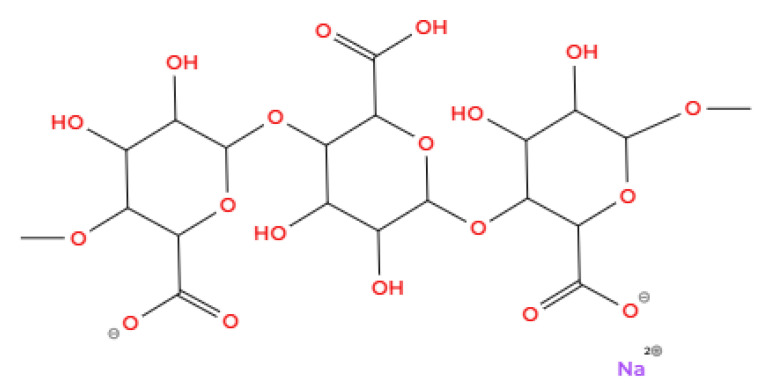
Sodium alginate (Illustration created with MolView.com; Adapted from: [106]).

Sodium alginate (NaC_6_H_7_O_6_) (see Figure 10), i.e., the sodium salt of alginic acid, is used in several industries, such as the pharmaceutical, textile, and food industries. Similar to gum Arabic, it is used in foods as a thickener, stabilizer, gelling agent, and emulsifier. Sodium alginate belongs to the group of carbohydrates derived from green waste [50], which comes from brown algae [57]. It is a wall material with a branched biopolymer structure [57]. It consists of two types of repetitive structural units, β-D-mannuronic acid and α-L-guluronic acid [112,113]. These units are connected by 1,4-glycosidic bonds and contain a carboxyl group at the C-6 position and a hydroxyl group at the C-2 position [112,113].

This wall material has many advantages and possibilities during microencapsulation. Sodium alginate is a white to yellowish-white, almost odorless, tasteless, stringy, or granular powder [108]. Like other alginates, it has many positive properties, such as its biodegradability, biocompatibility, low toxicity, and chemical versatility [50,57]. It is a stable and easy-to-store material [50]. Due to its large number of polar groups, it is highly hydrophilic and therefore easily soluble in water [113]. In addition, it is sustainable, simple, and cheap to produce, which makes it very suitable for encapsulation processes [50,57].

During microencapsulation by spray-drying, it has already been used in several researches as a wall material for tea and medicinal herbs. During the microencapsulation of green tea, Baltrusch et al. [50] found that sodium alginate had an exceptionally high yield and loading efficiency. It was also observed that the capsules had a thin coated wall, which, due to its nature, resulted in an extremely efficient release profile. Also, with the help of rheological analysis and experiments with UV light, it was established that this structure was able to provide adequate protection for the core material against environmental effects. Belščak-Cvitanović et al. [51] also observed that the gums, including sodium alginate, had the best color retention and the highest chlorophyll content in the product when examining green tea. Also, this wall material had the best physical properties, high EGCG content, and long-lasting dissolution/release profiles. This is what Ribeiro et al. [52] experienced during the encapsulation of elderberry, that the release was the slowest in the case of sodium alginate compared to modified chitosan and gum Arabic.

### 3.3. Inulin

**Figure 11:**
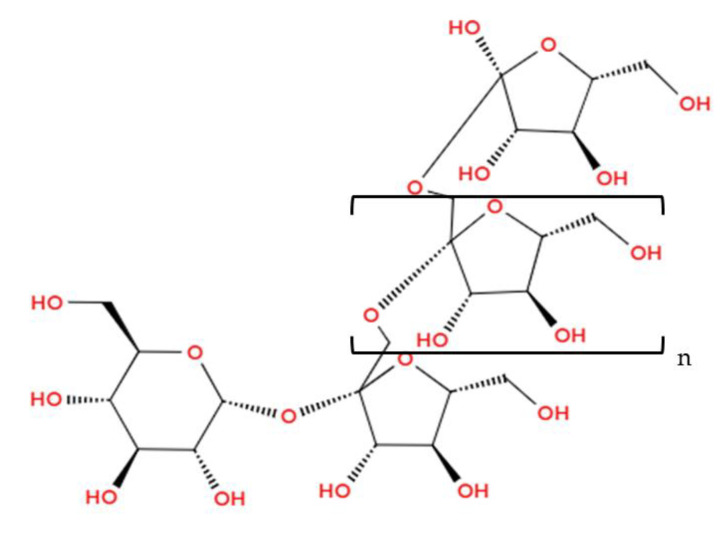
Inulin (Illustration created with MolView.com; Adapted from: [115]).

Inulin (C_6n_H_10n+2_O_5n_ + 1) is a chemically linear molecule of 2–60 fructose units composed of d-fructose monosaccharides and connected by β-(2,1)-glycosidic bonds [58,114]. As a variant, each molecule contains an elementary d-glucose unit (see Figure 11) [114]. Thanks to the β-configuration, inulin becomes stable against hydrolysis by human digestive enzymes; therefore, it is classified as an indigestible carbohydrate [114]. In addition to its prebiotic effect, it also has other beneficial effects on the human body, such as improved calcium bioavailability, as well as anticancer and immunomodulating properties [111].

Inulin is an important fructan found in approximately 45,000 plant species [114]. Examples of vegetables and fruits containing this substance are garlic, leeks, bananas, chicory, and yacon [114]. In industry, it is produced from chickpeas. It is currently considered a wall material that is available at a favorable price and in large quantities. In the course of research, it has so far been used for the microencapsulation of various oils (e.g., rosemary oil, corn oil, oregano oil) as a wall material. Still, it also has great potential for the encapsulation of teas and herbs, since many plants contain this substance. In the research of Vardanega et al. [8] with Brazilian ginseng, in the study of Çelik [55], and with black tea, inulin has already been successfully used as a carrier.

Inulin is a white, tasteless or slightly sweet, heat-stable wall substance. Its water solubility depends on the temperature and the size of the molecular chain [8]. Inulins with a higher degree of polymerization are generally less soluble at low temperatures [8]. De Barros Fernandes and colleagues [58] improved the wettability of the powders by adding inulin during the study of essential rosemary oil. During the research of Castel et al. [111], they found that, compared to gum Arabic (which is a widely used and widespread wall material), the moisture content increased with the addition of inulin, but the water activity was not affected. In addition, the addition of inulin to the product reduced brightness, redness, and yellowness, resulting in lighter-colored powders. Overall, inulin was found to be a good substitute for gum Arabic. When microencapsulating green tea, Belščak-Cvitanović et al. [51] found that inulin, modified starch, and carrageenan had the most favorable sensory properties; the reconstituted green tea microcapsules showed the least bitter and astringent green tea, as well as flavor. During his experiment, Çelik [55] established, based on the scanning electron images, that the powders were spherical during spray-drying, so the inulin encapsulated the extracts and provided adequate protection against environmental influences.

### 3.4. Whey Protein Isolate

Whey Protein Isolate consists of three different globular proteins: β-lactoglobulin, α-lactalbumin, and bovine serum albumin [62,93,96]. WPI is a by-product of cheese production, which is widely used in the food industry. From a health point of view, this substance has several advantages, as it has a high concentration of essential amino acids [93]. They are widely used as gelling and emulsifying agents due to their amphiphilic nature (thus containing both hydrophilic and hydrophobic parts), relatively large molecular size, and relatively high electrical charge [93,96].

On the other hand, WPI has other uses, as it has proven to be a good wall material for the microencapsulation of volatile compounds and has an excellent film-forming ability [62]. WPI can be an alternative option to traditional polysaccharide-based carriers, so it can offer an environmentally friendly solution to the industry [61,116]. WPI is a white, slightly cream-colored, tasteless, slightly sweet, and easily soluble wall material. It can be considered as a promising material for microencapsulation of various bioactive/hydrophobic compounds such as vitamins, carotenoids, and flavorings with improved stability and physicochemical properties [93]. At present, only a few studies have investigated it as a wall material for the encapsulation of tea and medicinal herbs.

During their research, Thi Aenh Dao et al. [78] found that WPI is effective as a wall material during the microencapsulation of green tea (e.g., increases the efficiency of dust recovery, and reduces adhesion), but its excessive application can already worsen the production process and the properties of the final product. Using higher amounts of WPI can increase the viscosity of the feed fluid, which will require a longer drying time and possibly cause clogging in the system. With a WPI content of more than 10%, the total polyphenol content in the powder is significantly reduced. In their research, Fang and Bhandari [60] compared the effects of maltodextrin and WPI during the atomization of bayberry juice. It was found that a small amount of protein (1%) was effective for microencapsulating a starting material with a high sugar content, while a large amount of maltodextrin (30%) was required to achieve this effect.

However, WPI has a few disadvantages. It is sensitive to environmental effects, especially pH, ionic strength, and high temperature, which can also cause interfacial protein denaturation [96].

## 4. Conclusions

The microencapsulation of tea and medicinal plant extracts still has many opportunities for research and development. The research so far mostly deals with the encapsulation of black and green tea, but there are still many types of medicinal herbs that have not been researched. Choosing the right encapsulation technique for a given raw material is a complex task. At present, most of the research in this area has been carried out with powder drying, and some research has been carried out with freeze-drying only. In both methods mentioned above, the properties of the finished products depend on several parameters. On the other hand, there is one parameter that greatly influences the properties of the instant powders used in both cases, and this is the carrier material used.

Wall materials can affect not only the internal properties, taste, and color of the final product, but also the structure, stability, solubility, viscosity, hygroscopic barrier, encapsulation efficiency, and protection of the core material of the capsules. When encapsulating tea and herbal extracts, the wall materials used can be divided into three large groups: carbohydrates, gums, and proteins. Maltodextrin and gum Arabic are the most researched and used wall materials for both freeze- and spray-drying. These excipients have many positive properties for encapsulation, such as good solubility, low viscosity, and protection of valuable substances. On the other hand, maltodextrin has a high carbohydrate content and is dependent on the quality of the material (DE), and gum Arabic is very expensive and limited in availability. That is why it is worth considering combining them with other alternative wall materials or replacing them altogether. So far, research has used modified starches such as beta-cyclodextrin and chitosan within the carbohydrates. Among the gums, sodium alginate receives attention in the literature, but the use of agar and Brea gum is also an area to be exploited. In addition, inulin and milk protein isolate have been used to spray-dry certain teas and herbal extracts. The testing of protein-based wall materials for microencapsulation of tea and herbal extracts is at a very rudimentary stage, but it would certainly be worth investigating skim milk, sodium caseinate, or lactose.

Since all wall materials have different properties and effects, the chemical composition of the given extract and the requirements for the final product must always be taken into account when using them. Since such diverse and different wall materials can be used, several researchers have already dealt with their combination in a certain ratio to combine different useful effects, but this topic still has many possibilities.

## Figures and Tables

**Table 1:** Core and wall materials for spray-drying technique. (The left column lists the references, indicating the studies published between 2007 and 2024. The middle column, ‘Core Material’, represents the teas or herbs used in the respective studies. The right column, ‘Wall Material’, details the auxiliary materials employed during the microencapsulation process).

REFERENCE	CORE MATERIAL	WALL MATERIAL
Alkali, 2017 [53]	Green tea (*Camellia sinensis* L.)	Chitosan, Gum Arabic, Maltodextrin
Baltaci et al., 2024 [54]	Rosehip fruits (*Rosa canina L.*)	There is none
Baltrusch et al., 2022 [50]	Green tea (*Camellia sinensis L.*)	Alginate, Carrageenan, Starch
Belščak-Cvitanović et al., 2015 [51]	Green tea (*Camellia sinensis L.*)	Acacia gum, Alginate, Carrageenan, Guar gum, Inulin, LBG, Modified starch, Oligofructose, Pectin, Pea proteins, Whey proteins, Xanthan
Braga et al., 2020 [30]	Pineapple (*Ananas comosus, Pérola var.*) with Spearmint (*Mentha spicata*) juice	Maltodextrin
Caliskan & Dirim, 2016 [36]	Sumac seed extract (*Rhus coriaria L.*)	Maltodextrin
Çelik, 2023 [55]	Green tea (*Camellia sinensis L.*) with Rockrose (Helianthemum nummularium)	Inulin
Chang et al., 2014 [56]	Shuang-Huang-Lian, Mulberry leaves (*Morus alba L.*), Lucid Ganoderma	Cyclodextrin, Leucin, Silicon dioxide
Chaumun et al., 2020 [57]	Bay laurel (*Laurus nobilis L.*)	Chitosan, Gum Arabic, Sodium alginate
de Barros Fernandes et al., 2014 [58]	Rosemary (*R. officinalis leaf oil*)	Gum Arabic, Inulin, Maltodextrin, Starch
Eroğlu et al., 2018 [15]	Roselle (*Hibiscus sabdariffa L.*) blended Rosehip fruits (*Rosa canina L.*)	Maltodextrin
Escobar-Avello et al., 2021 [59]	Grape cane extract (*V. vinifera L. cv. Pinot noir*)	Hydroxypropyl β-cyclodextrin, Maltodextrin
Fakher Dizaji et al., 2015 [12]	Peppermint (*Mentha piperita*)	There is none
Fang & Bhandari, 2012 [60]	Bayberry juice (*Myrica rubra*)	Maltodextrin, WPI (whey protein isolate)
Flores et al., 2014 [61]	Ripe rabbiteye (“Powderblue” cultivar) blueberries extract	WPI (whey protein isolate)
Hundre et al., 2015 [62]	*Vanilla Planifolia* (vanilin)	β-cyclodextrin, WPI (whey protein isolate)
Idham et al., 2012 [16]	Roselle (*Hibiscus sabdariffa L.*)	Gum Arabic, Maltodextrin, Starch
Insang et al., 2022 [63]	Mulberry leaves (*Morus alba L.*)	Maltodextrin
Kalajahi & Ghandiha, 2022 [64]	Nettle (*Urtica dioica L.*) extract	Maltodextrin
Kalogeropoulos et al., 2010 [65]	*Hypericum perforatum* (St John’s wort) extract	β-cyclodextrin
Kalušević et al., 2016 [9]	Peppermint (*Mentha piperita*), Chamomile (*Matricia chamomilla*), Wild thyme (*Thymus serpyllum*), Mountain germander (*Teucrium montanum*), Winter savory (*Satureja montana*), Yarrow (*Achillea millefolium*), Sage (*Salvia officinalis*), Lemon balm (*Melissa officinalis*), Centaurea (*Erythraea centaurium Pers.*), Wall germander (*Teucrium chamaedrys*), Nettle (*Urtica dioica*), Wormwood (*Artemisia absinthium*)	There is none
Kasapoğlu et al., 2024 [40]	*Rosa pimpinellifolia* fruit extract	Chitosan, Whey protein
Kraujalytė et al., 2016 [34]	Black tea (*Camellia sinensis L.*)	There is none
Krisetyadi & Hermansyah, 2021 [66]	Black tea (*Camellia sinensis L.*)	There is none
Latifi et al., 2020 [2]	Green tea (*Camellia sinensis L.*) with Cinnamon (*Cinnamomum verum*)	There is none
Lee et al., 2022 [10]	Chamomile (*Matricia chamomilla*)	Corn starch
Liu et al., 2021 [67]	Sweet Tea (*Lithocarpus litseifolius* [Hance] Chun)	There is none
Minh et al., 2019 [68]	*Fallopia multiflora*	Maltodextrin
Mora-Flórez et al., 2023 [5]	Peppermint (*Mentha piperita*) and Chamomile (*Matricia chamomilla*)	Maltodextrin, Sodium caseinate, Soy protein
Mourtzinos et al., 2011 [69]	Lemon balm (*Melissa officinalis L.*) leaf extract	β-cyclodextrin, Modified starch
Nguyen et al., 2022 [70]	Lotus and green tea (*Camellia sinensis L.*)	There is none
Nguyen et al., 2022 [17]	Roselle (*Hibiscus sabdariffa L.*)	Maltodextrin, Trehalose
Oliveira et al., 2023 [71]	*Hydrocotyle umbellata L.*	Maltodextrin, Silicon dioxide
Pandey & Manimehalai, 2014 [31]	Black tea (*Camellia sinensis L.*)	There is none
Pasrija et al., 2015 [24]	Green tea (*Camellia sinensis L.*)	β-cyclodextrin, Maltodextrin
Pudziuvelyte et al., 2019 [72]	Vietnamese balm (*Elsholtzia ciliata*)	β-cyclodextrin, Maltodextrin, Skim milk, Sodium caseinate
Ribeiro et al., 2019 [52]	Elderberry extract (*Sambucus Nigra L.*)	Chitosan, Gum Arabic, Sodium alginate
Şahin-Nadeem et al., 2011 [20]	Mountain tea (*Sideritis stricta*)	Gum Arabic, β-cyclodextrin, Maltodextrin
Şahin-Nadeem et al., 2013 [11]	Sage (*Salvia fruticosa Miller*)	Gum Arabic, β-cyclodextrin, Maltodextrin
Sarkhel et al., 2022 [3]	Mulberry leaves (*Morus alba L.*)	Maltodextrin
Secolin et al., 2017 [73]	Green tea (*Camellia sinensis L.*)	Lactose, Trehalose
Silva et al., 2018 [74]	Green tea (*Camellia sinensis L.*)	Cashew gum, Maltodextrin
Sinija & Mishra, 2008 [35]	Green tea (*Camellia sinensis L.*)	There is none
Sinija et al., 2007 [3]	Black tea (*Camellia sinensis L.*)	There is none
Someswararao & Srivastav, 2012 [75]	Black tea (*Camellia sinensis L.*)	There is none
Susantikarn & Donlao, 2016 [76]	Green tea (*Camellia sinensis L.*)	Maltodextrin
Tengse et al., 2017 [77]	Green tea (*Camellia sinensis L.*)	Maltodextrin
Thi Aenh Dao et al., 2021 [78]	Green tea (*Camellia sinensis L.*)	WPI (whey protein isolate)
Thuong et al., 2020 [18]	*Codonopsis javanica L.* root	Maltodextrin
Tran & Nguyen, 2018 [13]	Lemon balm (*Melissa officinalis L.*) leaf extract	Gum Arabic, Maltodextrin
Tülek et al., 2021 [14]	Lemon balm (*Melissa officinalis L*.) leaf extract	Maltodextrin
Vardanega et al., 2019 [8]	Brazilian ginseng (*Pfaffia glomerata*) root extracts	There is none
Vidović et al., 2014 [79]	*Satureja montana*	Maltodextrin
Vidović et al., 2019 [80]	Aronia or black chokeberry (*Aronia melanocarpa L.*)	Maltodextrin
Vladić et al., 2016 [6]	Yarrow (*Achillea millefolium*)	Maltodextrin
Vuong et al., 2013 [32]	Green tea (*Camellia sinensis L.*)	There is none
Wang et al., 2022 [81]	Pu-erh (*Camellia sinensis var. assamica*)	There is none
Zhang et al., 2020 [82]	Green tea (*Camellia sinensis L.*)	β-Glucosidase, β-Xylosidase
Zokti et al., 2016 [83]	Green tea (*Camellia sinensis L.*)	Gum Arabic, Maltodextrin, Chitosan

**Table 2:** Core and wall materials for freeze-drying technique. (The left column lists the references, indicating the studies published between 2007 and 2024. The middle column, ‘Core Material’, represents the teas or herbs used in the respective studies. The right column, ‘Wall Material’, details the auxiliary materials employed during the microencapsulation process).

REFERENCE	CORE MATERIAL	WALL MATERIAL
Baltaci et al., 2024 [54]	Rosehip fruits (*Rosa canina L.*)	There is none
Caliskan & Dirim, 2016 [36]	Sumac seed extract (*Rhus coriaria L.*)	Maltodextrin
Çelik, 2023 [55]	Green tea (*Camellia sinensis L.*) with Rockrose (Helianthemum nummularium)	Inulin
Fachinello et al., 2018 [26]	Green tea (*Camellia sinensis L.*)	There is none
Gui-yi et al., 2017 [90]	Pu’er tea, Gold Junmei tea, Tie Guanyin tea	There is none
Jo et al., 2003 [25]	Green tea (*Camellia sinensis L.*)	There is none
Khan et al., 2019 [93]	3,3′-Diindolylmethane (DIM)	WPI (whey protein isolate)
Kraujalytė et al., 2016 [34]	Black tea (*Camellia sinensis L.*)	There is none
Liu et al., 2021 [67]	Sweet Tea (*Lithocarpus litseifolius* [Hance] Chun)	There is none
Nguyen et al., 2022 [17]	Roselle (*Hibiscus sabdariffa L.*)	Maltodextrin, Trehalose
Pasrija et al., 2015 [24]	Green tea (*Camellia sinensis L.*)	β-cyclodextrin, Maltodextrin
Perera et al., 2015 [19]	Black tea (*Camellia sinensis L.*)	There is none
Pudziuvelyte et al., 2019 [72]	Vietnamese balm (*Elsholtzia ciliata*)	β-cyclodextrin, Maltodextrin, Skim milk, Sodium caseinate
Pudziuvelyte et al., 2020 [89]	Vietnamese balm (*Elsholtzia ciliata*)	Gum Arabic, Maltodextrin, Skim milk, Sodium caseinate
Ravichai & Muangrat 2019 [87]	Concentrated fermented Miang wastewater (CFMW)	Gum Arabic, Maltodextrin, Modified starch
Roshanak et al., 2016 [88]	Green tea (*Camellia sinensis L.*)	There is none
Sinija & Mishra, 2008 [35]	Green tea (*Camellia sinensis L.*)	There is none
Sinija et al., 2007 [3]	Black tea (*Camellia sinensis L.*)	There is none
Vardanega et al., 2019 [8]	Brazilian ginseng (*Pfaffia glomerata*) root extracts	There is none

## Data Availability

No new data were created or analyzed in this study. Data sharing is not applicable to this article.
